# Functional Characterisation of Microbial Communities Related to Black Stain Formation in Lascaux Cave

**DOI:** 10.1111/1758-2229.70112

**Published:** 2025-10-28

**Authors:** Zélia Bontemps, Danis Abrouk, Yvan Moënne‐Loccoz, Mylène Hugoni

**Affiliations:** ^1^ Université Claude Bernard Lyon 1, CNRS, INRAE, VetAgro Sup, UMR5557 Ecologie Microbienne Villeurbanne France; ^2^ Institut Universitaire de France (IUF) Paris France; ^3^ Université Claude Bernard Lyon 1, INSA Lyon, CNRS, UMR5240 Microbiologie Adaptation et Pathogénie Villeurbanne France

**Keywords:** biotransformations, metagenomics, microbial alterations, microbial interactions, palaeolithic caves

## Abstract

Anthropization of Palaeolithic caves may cause cave microbiota dysbiosis and promote the development of microbial stains on cave walls. In certain cases, chemical biocides have been used to mitigate rock alterations, but this may exacerbate microbiota unbalance. Here, we tested this model by metagenomics, using black stains that threaten art conservation in Lascaux Cave. Thus, we evidenced a wide range of microbial taxa differing between black stains and neighbouring unmarked surfaces. Genes for synthesis of melanin and carotenoid pigments were more prevalent in black stains and were identified in reconstructed genomes for fungi (as expected) and bacteria. The presence of genes for degradation of aromatic compounds supports the hypothesis that recycling of chemical biocides favoured melanin‐producing microorganisms. These findings extend previous predictions by revealing a wider range of microorganisms, potential biotransformations favouring pigment synthesis, as well as microbial interactions influencing microbial dynamics during cave wall alterations.

## Graphical abstract

Excessive anthropization can lead to black stain alterations on cave walls. The present work reports the first metagenomic analysis of black stains threatening artwork in Lascaux Cave. Findings show new microbial taxa and genetic potential associated with black stains. Understanding black stain formation is useful for cave conservation strategies.

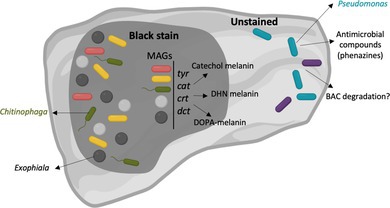

## 1 Introduction

Microorganisms drive biodeterioration processes that may affect parietal art in caves subjected to excessive tourism (Martin‐Sanchez et al. 2015), as found in Lascaux (France), where anthropization has changed environmental and microbiota conditions (Bastian et al. 2009; Bontemps et al. 2022). In Lascaux, chemical biocides including benzalkonium chloride (BAC) were used to treat microbial alterations, which promoted further microbiota unbalance (Martin‐Sanchez et al. 2015).

In Lascaux, metagenomics of recent alterations termed dark zones evidenced enhanced pigment biosynthesis potential compared with healthy surfaces nearby, whereas genes for degradation of aromatic compounds (such as BAC) were prevalent in both conditions (Bontemps et al. 2024). However, black‐stain alterations (i.e., stains of black colour documented in relation to black melanized fungi) are more widespread than dark zones (i.e., zones of darker shade than on unmarked surfaces around, but without the black colour of black stains, (Bontemps et al. 2022)), and they are documented also in the Cave of Bats (Spain; Urzì et al. 2010), Etruscan tombs (Italy; Isola et al. 2021) and Driny Cave (Slovakia; Ogórek et al. 2016). In Lascaux, they are typically attributed to melanin‐producing pigmented fungi such as *Ochroconis lascauxensis* (Martin‐Sanchez et al. 2012), based on isolation of such fungi coupled with cloning‐sequencing or metabarcoding approaches (Martin‐Sanchez et al. 2015; Alonso et al. 2023).

The assessments to date point to a likely scenario for black stain formation (Bontemps et al. 2022; Martin‐Sanchez et al. 2012). However, does microbial diversity documented by PCR‐based metabarcoding correctly reflect the microbiota particularities of black stains? Melanin has been evidenced in fungal isolates, but which biosynthetic processes are involved in situ, and in which microorganisms? Which other metabolic pathways might also be implicated to yield substrates for melanin synthesis, and what is the contribution of past chemical treatments? Certain microorganisms (i.e., *Pseudomonas*) isolated outside black stains inhibit pigmented fungi in vitro (Alonso et al. 2023), but what is the significance of microbial inhibition to control black stain expansion?

Here, we tested the hypotheses that (i) metabarcoding and metagenomics would give convergent diversity findings, (ii) genetic pathways for melanin synthesis would be prevalent in black stains, (iii) melanin synthesis is potentially fueled by degradation products of the chemical biocides previously used and (iv) pathways involved in the synthesis of antimicrobials should occur, particularly outside black stains. Thus, we investigated microbial communities in black stains versus unmarked surfaces by metagenomics coupled with genome‐resolved metagenomics (i.e., metagenome‐assembled genomes).

## 2 Materials and Methods

### 2.1 Sampling, DNA Extraction and Quantitative PCR

Lascaux Cave is located in Périgord, France (N 45°03′13.087″ and E 1°10′12.362″). We focused on the Nave room, where black stains started in 2006 (Figure S1). Stains that developed from January 2019 on were sampled in 2020 (Figure S1). Three black stains and three unmarked limestone surfaces nearby (i.e., three biological replicates each; the six spots were 5–15 cm from one another) were sampled with sterile scalpels (using < 1 cm^2^) and placed into liquid nitrogen. Total DNA was extracted using the FastDNA SPIN Kit for Soil (MP Biomedicals, Illkirch, France) (see Supporting Information). The numbers of bacterial 16S rRNA genes, archaeal 16S rRNA genes, and microeukaryotic 18S rRNA genes were estimated by quantitative PCR (qPCR) (Supporting Information).

### 2.2 Metabarcoding

Microbial composition of altered and unmarked surfaces in Lascaux Cave was determined using metabarcoding with Illumina MiSeq (2 × 300 bp, chemistry v3), as described in Bontemps et al. (2023) (Supporting Information). Briefly, reads were quality filtered (i.e., no Ns, expected size) and clustered into OTUs using SWARM (Mahé et al. 2014), which uses a local clustering threshold (rather than a global clustering threshold) and an aggregation distance of 3.0. Chimeric, low abundance (< 0.005%) and contaminant OTUs were discarded from the dataset. Taxonomic affiliation was performed using both RDP Classifier and BLASTn (Zhang and Madden 1997) against the 138.1 SILVA database (Quast et al. 2013) for Bacteria, Archaea and Eukaryotes, which was automated in the FROGS pipeline (Escudié et al. 2018). Samples were randomly resampled to the lowest number of sequences retrieved per sample, that is 127,994, 11,353 and 210,384 sequences for bacterial 16S rRNA genes, archaeal 16S rRNA genes and eukaryotic 18S rRNA genes, respectively (Table S1).

### 2.3 Metagenomic Library Preparation, Sequencing and Analysis

Three metagenomic libraries were constructed for limestone samples and three others for black stains, using NEBNext Ultra DNA Library Prep Kit (fragment size ~350 bp, Illumina, San Diego, USA) according to the manufacturer's recommendations. Sequencing was performed by Genewiz company (Germany) using Illumina NovaSeq 6000 system 2 × 150 bp. Adapter sequences were removed from the metagenomic data on the instrument and sequence data were exported in FASTQ files. Datasets were quality filtered using Trimmomatic (Bolger et al. 2014) with default settings. All metagenomes were then pooled and co‐assembled using MEGAHIT (Li et al. 2015). Reads were mapped against contigs using Bowtie to estimate coverage (Langmead and Salzberg 2012). Assembly quality was evaluated using *prinseq* (Schmieder and Edwards 2011) and presented in Table S2. Gene prediction was achieved using Prodigal and rRNA was detected using *barrnap* (https://github.com/tseemann/barrnap), before classification using RDP classifier (Wang et al. 2007). The latest versions of KEGG (Kanehisa and Goto 2000), MetaCyc (Karp et al. 2002) and COG (Galperin et al. 2021) databases (April 2022) were used for functional annotation. To allow sample comparisons, metagenomes were normalised to the lowest number of sequences (151,801,037 reads; Table S1).

In addition, reconstruction of genomic bins was performed, using the two binning algorithms MetaBAT‐2 and Maxbin 2.0 (Kang et al. 2019; Wu et al. 2014) and contigs longer than 2000 bp. Redundant bins obtained from both binning algorithms were identified and removed using DAS Tool (Sieber et al. 2018). The completeness and contamination level of the combined genomic bins were then evaluated using CheckM for prokaryotic bins (Parks et al. 2015) and Busco for microeukaryotic bins (Simão et al. 2015). Only bins with a contamination level under 5% and completeness above 75% were analysed and represented using ‘*circlize’* package in R software (Gu 2022). Genetic composition of bins was then explored using KEGG (Kanehisa and Goto 2000), MetaCyc (Karp et al. 2002) and COG (Galperin et al. 2021) databases, based on genes identified in the co‐assembly. Metabolic pathways in which 75% of the genes were present were considered and represented using ‘*circlize’* and ‘*CircleHeatmap’* packages in R software (Gu 2022). A total of 31 bins with > 75% completeness and < 5% contamination levels were reconstructed from metagenomes of Lascaux Cave (Table S3). The most represented lineages in reconstructed bins were Alphaproteobacteria (9 different bins), Actinomycetota (4 bins), Bacteroidota (4 bins) and Gammaproteobacteria (4 bins) (Figure S2).

### 2.4 Statistical Analyses

Rarefaction curves, α‐diversity and β‐diversity analyses were done as described (Bontemps et al. 2023). Student *t*‐tests and Wilcoxon tests were performed using ‘*stats’* package in R (Oksanen et al. 2024), to test differences (*p* < 0.05) in diversity index and metabolic classes between different sampling zones (unmarked surfaces vs. dark zones). The radar plot was performed using ‘*plotly’* package (https://plotly‐r.com). Enrichment plot was made using the ‘*enrichplot’* (Yu and Hu 2022) and ‘*fcros’* (https://r‐packages.io/packages/fcros/fcrosMod) packages in *R* and *p* value was estimated by a hypergeometric distribution.

## 3 Results and Discussion

### 3.1 Community Composition

**FIGURE 1:**
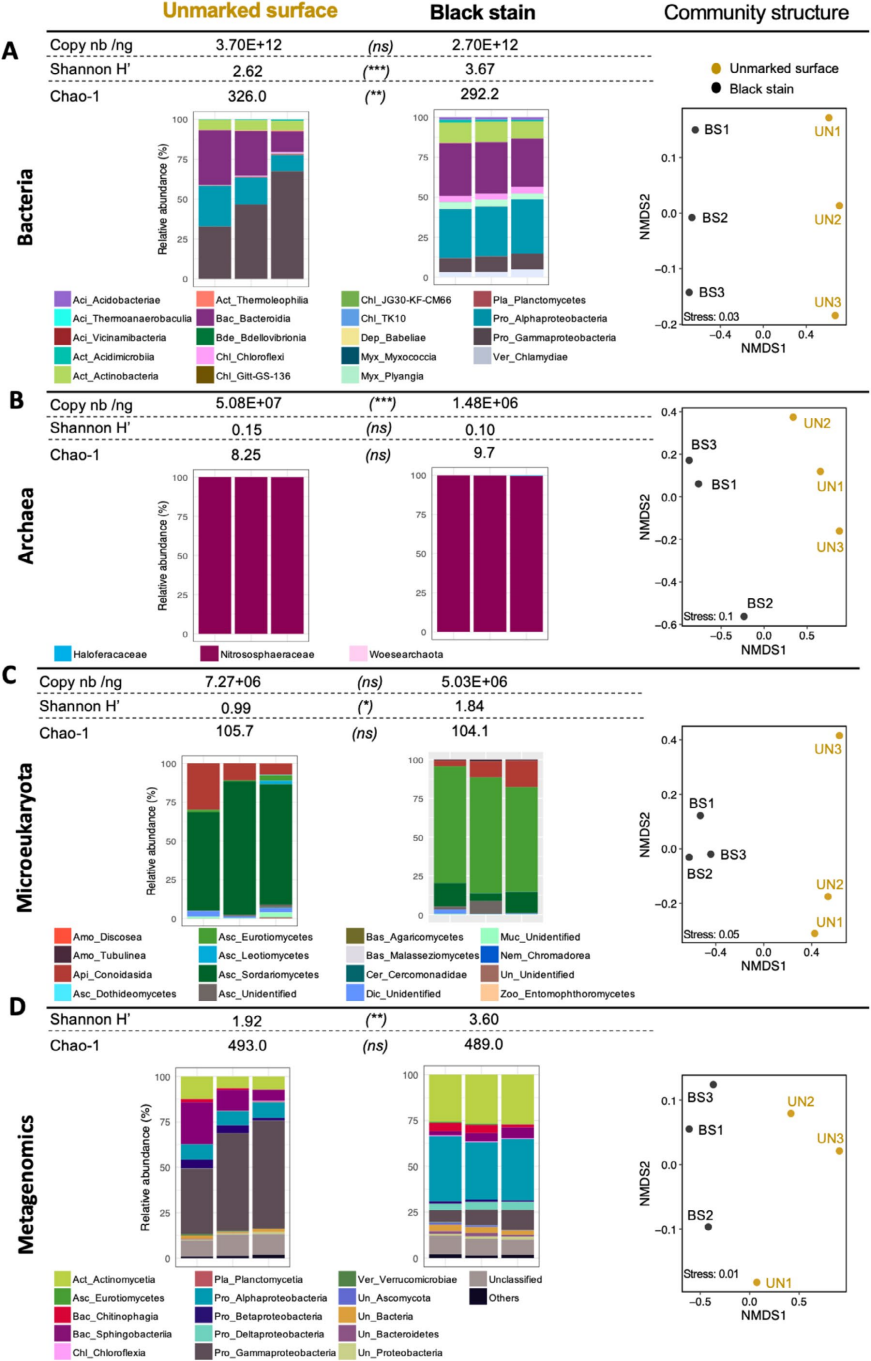
Abundance, diversity and community structure in unmarked surfaces and black stains of Lascaux Cave. Data are shown for bacteria (A), archaea (B), microeukaryotes (C) and the total community (D) by shotgun metagenomics. Microbial abundance (qPCR) is shown in A–C, diversity (Shannon H′ and Chao‐1 indices), the distribution of the most abundant classes (i.e., consisting in > 0.01% of total normalised sequences; samples UN1 to UN3 and BS1 to BS3 from left to right) and Non‐metric MultiDimensional Scaling (NMDS; based on Bray–Curtis dissimilarity) of microbial communities in A–D. Copy nb/ng: Gene copy number/ng ADNg in sample. Significance of Student *t*‐tests: (ns), not‐significant; (*), *p* < 0.05; (**), *p* < 0.01; (***), *p* < 0.005. Aci, Acidobacteriota; Act, Actinomycetota; Amo, Amoebozoa; Api, Apicomplexa; Asc, Ascomycota; Bac, Bacteroidota; Bas, Basidiomycota; Bde, Bdellovibrio; Cer, Cercozoa; Chl, Chloroflexota; Dep, Dependentiae; Dic, Dicosoa; Muc, Mucoromycota; Myx, Myxcoccota; Nem, Nematozoa; Pla, Planctomycetota; Pro, Pseudomonadota (formerly Proteobacteria); Un, Unidentified; Ver, Verrucomicrobiota; Zoo, Zoopagomycota.

Metagenomics expanded the range of bacterial diversity documented in black stains and unmarked surfaces, as Actinomycetia, Sphingobacteria, Chitinophagia, Betaproteobacteria were identified only by metagenomics (Figure 1). Black stains and unmarked surfaces differed, confirming metabarcoding data also showing those differences (Figure 1).

The highest variation in relative abundance of classes was found for bacteria (85.5% of metagenomic sequences; Table S1), that is Gammaproteobacteria (−42.8% in black stains compared with unmarked surfaces), Alphaproteobacteria (+24.9%), Actinomycetia (+17.0%), Sphingobacteriia (−7.3%), Deltaproteobacteria (+4.0%) and Chitinophagia (+2.6%), followed by fungi (0.7% of metagenomic sequences), that is Eurotiomycetes (+0.47%) and Sordariomycetes (−0.4%). Among Gammaproteobacteria, *Pseudomonas* counter‐selection was already documented (Alonso et al. 2023). We obtained one *Pseudomonas* bin from black stain (with 209 metabolic pathways) and another one from unmarked surface (118 pathways) (Figures S2 and S3; Table S3), pointing to contrasted ecological adaptations. Community modifications took place without major change in size, based on qPCR of bacterial 16S rRNA genes (2.7 ± 4.3 × 10^12^ and 3.7 ± 2.6 × 10^12^ gene copies/ng total DNA; *p* = 0.13; Figure 1A) and microeukaryotic 18S rRNA genes (5.0 ± 3.6 × 10^6^ and 7.3 ± 2.4 × 10^6^ copies/ng total DNA; *p* = 0.42; Figure 1C) for respectively black stains and unmarked surfaces, but archaeal 16S rRNA genes showed lower copy number (*p* = 0.001) in black stains (1.5 ± 1.8 × 10^6^ vs. 5.1 ± 2.8 × 10^7^ copies/ng total DNA; Figure 1B).

### 3.2 Melanin Synthesis Potential

**FIGURE 2:**
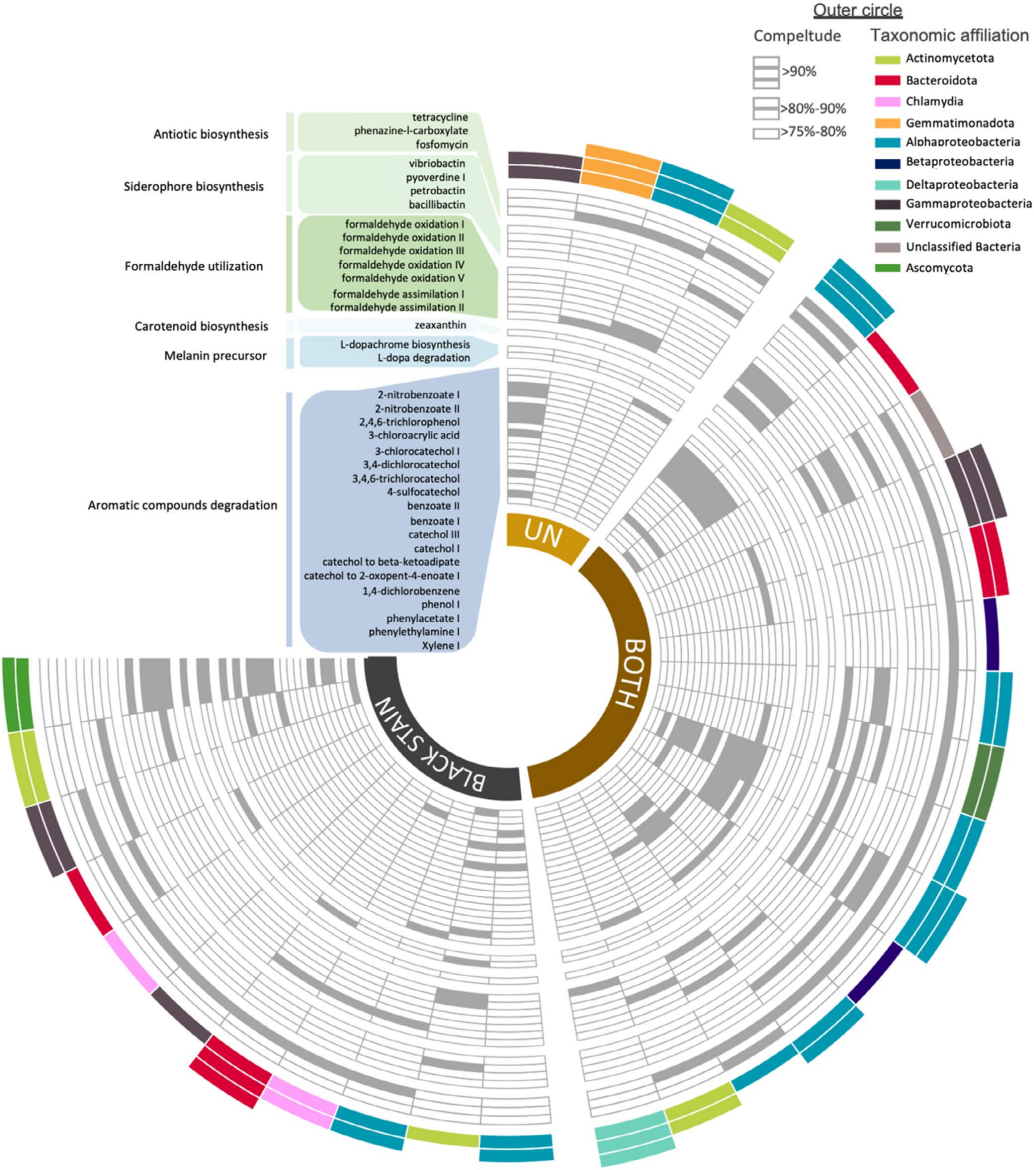
Key metabolic pathways identified in reconstructed genomic bins. Six metabolic classes are represented: Antibiotic biosynthesis, Siderophore biosynthesis, Formaldehyde utilisation, Carotenoid biosynthesis, Melanin precursor metabolism and Aromatic compounds degradation. Colours in the outer circle represent the taxonomic affiliation of the bins. Details of genomic bins (ID, completeness, contamination, taxonomy and coverage) are presented in Table S4.

The black shade of the stains is attributed to melanin from pigmented fungi (*Ochroconis*, *Exophiala, Acremonium*, etc.) (De la Rosa et al. 2017; Martin‐Sanchez et al. 2012). Analysis of the 9822 genes (KEGG orthologues) evidenced by metagenomics showed that metabolic genes or pathways for synthesis of melanin (or its precursors) were more prevalent in black stains, as found with dark‐zone alterations (Bontemps et al. 2024). Specifically, *tyr* genes (tyroninase) were identified only in black stains and in bins from black stains (Figures 2 and S2; Table S4).

The L‐3,4‐hydroxyphenylalanine (L‐dopa) degradation pathway was found in four bins affiliated to bacteria (Actinomycetia class, Bacteroidota phylum and *Chitinophaga* genus) or the fungus *Exophiala*. L‐Tyrosine is converted to L‐dopa and L‐dopa to dopaquinone, dopaquinone spontaneously forming the eumelanin precursor L‐dopachrome (Solano 2014). Accordingly, melanins were evidenced in black stains by chemical analysis (performed as in Bontemps et al. 2024). Melanins can be produced both by bacteria and fungi (Ischia et al. 2013; De la Rosa et al. 2017), but we reveal here that bacteria in Lascaux's black stains have the potential to synthesise melanin. Also, the melanin‐producing fungus *Exophiala* was more abundant in black stains than in unmarked surfaces based on 18S rRNA metabarcoding. Overall, this strengthens the hypothesis that genetic pathways for melanin synthesis are prevalent in black stains, thereby extending the range of microorganisms potentially involved.

### 3.3 Biocide Degradation Potential

**FIGURE 3:**
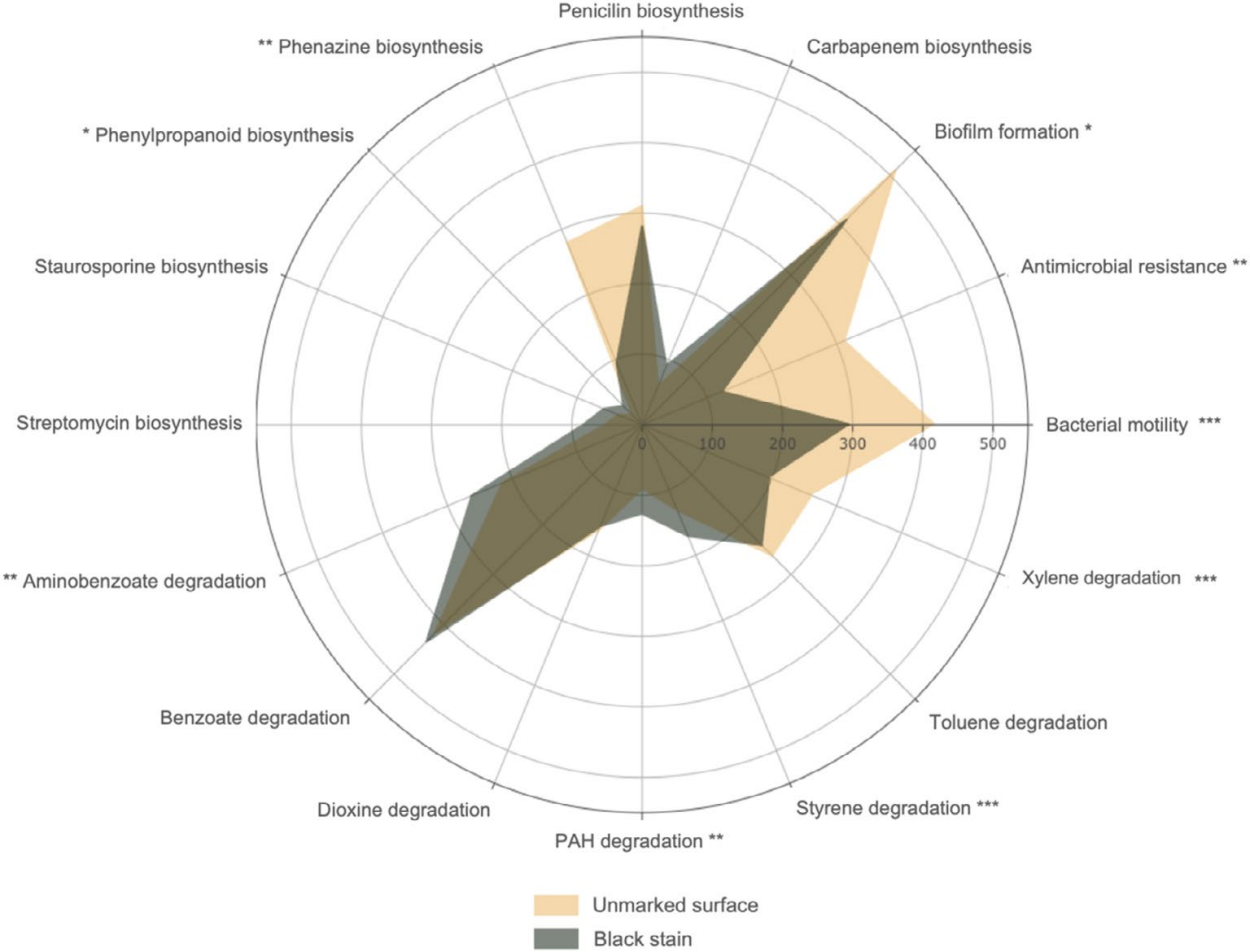
Relative proportion of metabolic pathways from unmarked surfaces (yellow) and black stains (grey) of Lascaux Cave, assessed using a matrix that was square‐root‐transformed to minimise the impact of highly‐dominant functional classes. Significance of Wilcoxon tests: ‐, not‐significant; *p < 0.05; **p < 0.01; ***p < 0.005. PAH: polycyclic aromatic hydrocarbons.

Previous studies on Lascaux isolates have pointed out that melanin synthesis was likely stimulated by the degradation of aromatic biocides, which had probably favoured melanin‐producing microorganisms. As in dark zones (Bontemps et al. 2024), genes and pathways for the degradation of aromatic compounds were found inside and outside black stains in the present work. They were more prevalent in black stains and concerned for example genes for the degradation of aminobenzoate (69,113 genes in stains vs. 41,386 outside; *p* = 0.003), polycyclic aromatic hydrocarbons (15,777 vs. 8099 genes; *p* = 0.004) and styrene (29,052 vs. 12,713 genes; *p* = 0.0002) (Figure 3), which may be useful for the metabolism of the melanin precursor catechol.

When considering bins, genes for xylene degradation were only found in bins (two affiliated with Actinobacteriota and one with Ascomycota) from black stains (Figure S2). Genes *xylEFJK* and *todGHI* of the catechol degradation pathway were identified only in Alphaproteobacteria (*Hyphomicrobium*) and Actinobacteriota bins from black stains. The catechol degradation III pathway and catechol degradation to β‐ketoadipate pathway were found in three bins, that is two Alphaproteobacteria reconstructed from both surface conditions (*Phyllobacterium* and *Bradyrhizobium*) and one Ascomycota from black stains (Figure 2). The catechol meta‐cleavage pathway converts benzoate into catechol, whereas the catechol ortho‐cleavage pathway produces succinyl‐CoA and acetyl‐CoA, necessary for biosynthesis of DHN‐melanin (Solano 2014). The formation of catechol‐melanin can involve a disproportionation reaction between catechol and a benzoquinone (produced by catechol oxidase or degradation of 2,4,6‐trichlorotoluene, as observed here in the *Pseudomonas spelaei* bin), generating radical semi‐quinones. Spontaneous reactions generate aromatic biphenolic dimers or diphenylene‐dioxide‐2,3‐quinone, resulting in a catechol‐melanin polymer (Nicolaus 1968). Therefore, the hypothesis that genes for aromatic compound degradation would be well present both in and outside black stains proved correct.

### 3.4 Antagonistic Potential

We considered genes and metabolic pathways involved in the synthesis of antimicrobial compounds that might inhibit pigmented microorganisms on unmarked surfaces. Phenazine biosynthesis genes were less abundant in black stains than on unmarked surfaces (8950 vs. 77,907; *p* = 0.009) (Figure 3), but phenylpropanoid biosynthesis was more represented in black stains (1410 vs. 304 genes; *p* = 0.04). Pathways associated with the biosynthesis of antibiotics or pyoverdine were detected in > 20 bins across both surface conditions (Figures 2 and S2). Therefore, in contrast to our hypothesis, biosynthesis pathways for antimicrobials were found not only in bins from unmarked surfaces but also in bins from black stains.

## 4 Conclusions

Here, metagenomics revealed that (i) microbial community differed between black stains and unmarked surfaces, including for taxa poorly recovered by PCR, (ii) pigmented microorganisms included also bacteria, (iii) the genetic potential existed to convert biocide residues into melanin and (iv) microbial dynamics might be influenced by antimicrobial compounds on both surfaces. These features present similarities with findings obtained from dark‐zone alterations (Bontemps et al. 2024), suggesting that subtle variations in microbial diversity, genetic potential for pigment synthesis, biocide residue degradation and antagonism ability can lead to contrasted types of surface alterations. Understanding the formation of the various alterations due to overtourism is crucial for protecting Palaeolithic heritages, in Lascaux and other anthropized caves.

## Author Contributions

Z.B. contributed to sampling, acquired data, interpreted results, wrote the manuscript, and prepared figures and tables; D.A. acquired data; Y.M.‐L. obtained funding, managed the project, designed the experiments, contributed to sampling, interpreted results, and revised the manuscript; M.H. interpreted results and revised the manuscript.

## Conflicts of Interest

The authors declare no conflicts of interest.

## Supporting information


**Data S1.**emi470112‐sup‐0001‐Supinfo.docx.

## Data Availability

The data that support the findings of this study (metabarcoding and metagenomics) are openly available in the NCBI public database under Bioproject PRJNA860536. Considering metagenomic data, bin files are available in FigShare https://doi.org/10.6084/m9.figshare.20626854.v1.
