# Supplementary material for: Functional Characterisation of Microbial Communities Related to Black Stain Formation in Lascaux Cave
Source: Environ Microbiol Rep. 2025 Oct 28;17(6):e70112. doi: 10.1111/1758-2229.70112 (PMC12566190; doi:10.1111/1758-2229.70112)
Supplement: Supplementary file 1 — Data S1.emi470112‐sup‐0001‐Supinfo.docx. [file EMI4-17-e70112-s001.docx]

**Supplementary File 1.**

**Functional characterization of microbial communities related to black stain formation in Lascaux Cave**

Zélia Bontemps^1^, Danis Abrouk^1^, Yvan Moënne-Loccoz^1,2^, Mylène Hugoni^2,3*^

^1^ Université Claude Bernard Lyon 1, CNRS, INRAE, VetAgro Sup, UMR5557 Ecologie Microbienne, F-69622 Villeurbanne, France

^2^ Institut Universitaire de France (IUF), Paris, France

^3^ Université Claude Bernard Lyon 1, INSA Lyon, CNRS, UMR5240 Microbiologie Adaptation et Pathogénie, F-69621 Villeurbanne, France

*Corresponding author: [mylene.hugoni@univ-lyon1.fr](mailto:mylene.hugoni@univ-lyon1.fr)

**Keywords:** Paleolithic caves, microbial alterations, microbial interactions, metagenomics, biotransformations

**Authors ORCID**

Mylène Hugoni: 0000-0002-2430-1057​

Yvan Moënne-Loccoz: 0000-0002-9817-1953

Zélia Bontemps: 0009-0002-6785-9197

**Supplementary Materials and Methods**

**DNA extraction**

Total DNA was extracted using the FastDNA SPIN Kit For Soil (MP Biomedicals, Illkirch, France) with modification of the FastPrep step, i.e. 80 s at a speed setting of 6.0 m/s and 15 min at 4 °C for centrifugation. The elution step was achieved using 50 µl elution buffer for each sample. The DNA concentrations were quantified using the Qubit dsDNA HS Assay Kit (Invitrogen, Carlsbad, USA) following the manufacturer’s instructions. The DNA extracts were stored at -20 °C until library preparation.

**Quantitative PCR**

Bacterial 16S rRNA genes, archaeal 16S rRNA genes and microeukaryotic 18S rRNA genes were monitored by quantitative PCR (qPCR). It was performed using primers 519F (5’-CCGTCAATTCMTTTRAGTTT-3’) / 907R (5’-AAGGAAGGCAGCAGGCG-3’) (Laiz et al., 2003), 787F (5’- ATTAGATACCCSBGTAGTCC-3’) / 1059R (5’- GCCATGCACCWCCTCT-3’) (Nehmé et al., 2009), and Euk345F (5’- AGGAAGGCAGCAGGCG-3’) / Euk499R (5’-CACCAGACTTGCCCTCYAAT-3’) (Zhu et al., 2019), respectively. Briefly, qPCR assays were conducted using 10 µl of LightCycler 480 SYBR Green I Master mix (Roche Diagnostics, Meylan, France), 2 µl of sample DNA, 2 µl (final concentration 0.3 µM) of each primer in a final volume of 20 µl, in the thermocycler CFX-96 Connect (Bio-Rad, Hercules, United States). qPCR programs for bacteria and eukaryotes consisted in an initial denaturation at 95^◦^C for 10 min, followed by 40 cycles of 15 s denaturation at 95^◦^C, hybridization 60 s at 63^◦^C (16S) or 15 s at 60^◦^C (18S), and 30 s (16S) or 15 s (18S) elongation at 72^◦^C. qPCR for archaea was done with an initial denaturation at 37^◦^C for 10 min and 95^◦^C for 15 min, followed by 45 cycles of 15 s denaturation at 95^◦^C and 60 s hybridization at 60^◦^C. Standard curves for targeted genes (bacterial 16S rRNA gene, archaeal 16S RNA gene and microeukaryotic 18S rRNA gene) were generated from a mix of plasmids representative of the environment studied (NucleoSpin Plasmid, Macherey-Nagel, Hoerdt, France). Melting curve calculation and Tm determination were done using the Tm Calling Analysis module of CFX Maestro Software v 2.3 (Bio-Rad).

**Metabarcoding: Illumina MiSeq amplicon libraries, sequencing and analysis**

Metabarcoding of bacterial 16S rRNA genes, archaeal 16S rRNA genes and eukaryotic 18S rRNA genes was performed using primers targeting bacterial V3-V4 region (341F and 805R; 550 bp product) (Herlemann et al., 2011), archaeal V3-V4 region (515F and 915R; 420 bp product) (Herfort et al., 2009) and V2 eukaryotic region (18S_0067a_deg and 18S_NSR399; 350 bp product) (Dollive et al., 2012), respectively. All primers were tagged with the Illumina adapter sequences (TCG TCG GCA GCG TCA GAT GTG TAT AAG AGA CAG and GTC TCG TGG GCT CGG AGA TGT GTA TAA GAG ACA G) allowing the construction of amplicon libraries by a two-step PCR. All PCR reactions were carried out following Bontemps et al. (2023). High-throughput sequencing was achieved after pooling PCR triplicates using Illumina MiSeq (2 × 300 bp, chemistry v3), and was performed by Microsynth (Balgach, Switzerland), aiming for each sample at 40,000 sequences for bacterial 16S rRNA gene and eukaryotic 18S rRNA gene, and 70,000 sequences for archaeal 16S rRNA gene (to compensate for some primer aspecificity).

For each dataset, paired-end reads were demultiplexed according to exact match adaptors (removed) and reads were merged with a maximum of 10% mismatches in the overlap region using FLASh (Magoč and Salzberg, 2011). Denoising procedures were carried out by discarding reads without the expected length (200-500 bp) or containing any ambiguous bases (N). After dereplication of sequences, clusterisation of sequences into OTUs was performed using SWARM (Mahé et al., 2014), which uses a local clustering threshold (rather than a global clustering threshold) and an aggregation distance of 3.0 for identification of operational taxonomic units (OTUs). Chimeric sequences were discarded using VSEARCH (Rognes et al., 2016). OTUs with low abundance (≤ 0.005%) were filtered, i.e. keeping OTUs representing at least 0.005% of the dataset, along with singletons that were also removed from the datasets. Taxonomic affiliation was performed using both RDP Classifier and BLASTn (Zhang and Madden, 1997) against the 138.1 SILVA database (Quast et al., 2013) for Bacteria, Archaea and Eukaryotes, which was automated in the FROGS pipeline (Escudié et al., 2018). Contaminant OTUs identified from the negative control samples (blanks) were removed and the eukaryotic dataset was manually curated for metazoan sequences (data not shown). Finally, samples were randomly resampled to lowest number of sequences retrieved per sample, that is 127,994, 11,353 and 210,384 sequences for bacterial, archaeal and microeukaryotic datasets, respectively, to allow comparisons between samples (Table S2). Raw sequences were deposited in the NCBI public database under Bioproject PRJNA860536.

**Statistics**

Rarefaction curves were calculated to assess sequencing efficacy, using Paleontological Statistics (PAST) software v4.02 (Hammer et al., 2001). OTU richness and diversity were estimated using Chao 1 index (Chao, 1987) and Shannon H’ (Shannon, 1948). Communities were primarily compared with Non-metric MultiDimensional Scaling (NMDS) based on the Bray-Curtis dissimilarity matrix, using ‘*vegan’* package in R v4.0.2 (Oksanen et al., 2020; R Core Team, 2020). The procedure computes a stress value, which measures the difference between the ranks on the ordination configuration and the ranks in the original dissimilarity matrix for each replicate. Stress values below 0.1 are ideal, those below 0.2 acceptable (especially if close to 0.1), while values above 0.2 point to limited interpretation potential (Clarke, 1993). Analysis of similarity (ANOSIM) was conducted using the ‘*vegan’* package in R, to test differences (*P* < 0.05) in overall community composition between different sampling zones (unmarked surfaces vs dark zones) and to further confirm the results observed in the NMDS plot.

**References**

Bontemps Z, Prigent-Combaret C, Guillmot A, Hugoni M, Moënne-Loccoz Y. Dark-zone alterations expand throughout Paleolithic Lascaux Cave despite spatial heterogeneity of the cave microbiome. 2023. *Environmental Microbiome* 18:31. <https://doi.org/10.1186/s40793-023-> 00488-8

Chao A. 1987. Estimating the population size for capture-recapture data with unequal catchability. *Biometrics* 43:783–791. doi:10.2307/2531532

Clarke KR. 1993. Non-parametric multivariate analyses of changes in community structure. *Australian Journal of Ecology* 18:117–143. doi:https://doi.org/10.1111/j.1442-9993.1993.tb00438.x

Dollive S, Peterfreund GL, Sherrill-Mix S, Bittinger K, Sinha R, Hoffmann C, Nabel CS, Hill DA, Artis D, Bachman MA, Custers-Allen R, Grunberg S, Wu GD, Lewis JD, Bushman FD. 2012. A tool kit for quantifying eukaryotic rRNA gene sequences from human microbiome samples. *Genome Biology* 13:R60. doi:10.1186/gb-2012-13-7-r60

Escudié F, Auer L, Bernard M, Mariadassou M, Cauquil L, Vidal K, Maman S, Hernandez-Raquet G, Combes S, Pascal G. 2018. FROGS: Find, Rapidly, OTUs with Galaxy Solution. *Bioinformatics* 34:1287–1294. doi:10.1093/bioinformatics/btx791

Hammer Ø, Harper DA, Ryan P. 2001. PAST: paleontological statistics software package for education and data analysis. *Palaeontologia Electronica* 4:1.

Herfort L, Kim J-H, Coolen MJL, Abbas B, Schouten S, Herndl GJ, Damsté JSS. 2009. Diversity of Archaea and detection of crenarchaeotal *amoA* genes in the rivers Rhine and Têt. *Aquatic Microbial Ecology* 55:189–201. doi:10.3354/ame01294

Herlemann DP, Labrenz M, Jürgens K, Bertilsson S, Waniek JJ, Andersson AF. 2011. Transitions in bacterial communities along the 2000 km salinity gradient of the Baltic Sea. *The ISME Journal* 5:1571–1579. doi:10.1038/ismej.2011.41

Laiz L, Piñar G, Lubitz W, Saiz-Jimenez C. 2003. Monitoring the colonization of monuments by bacteria: cultivation versus molecular methods. *Environmental Microbiology* 5:72–74. doi:10.1046/j.1462-2920.2003.00381.x

Magoč T, Salzberg SL. 2011. FLASH: fast length adjustment of short reads to improve genome assemblies. *Bioinformatics* 27:2957–2963. doi:10.1093/bioinformatics/btr507

Mahé F, Rognes T, Quince C, de Vargas C, Dunthorn M. 2014. Swarm: robust and fast clustering method for amplicon-based studies. *PeerJ* 2:e593. doi:10.7717/peerj.593

Nehmé B, Gilbert Y, Létourneau V, Forster RJ, Veillette M, Villemur R, Duchaine C. 2009. Culture-independent characterization of archaeal biodiversity in swine confinement building bioaerosols. *Applied and Environmental Microbiology* 75:5445–5450. doi:10.1128/AEM.00726-09

Oksanen J, Blanchet FG, Friendly M, Kindt R, Legendre P, McGlinn D, Minchin PR, O’Hara RB, Simpson GL, Solymos P, Stevens MHH, Szoecs E, Wagner H. 2020. vegan: Community Ecology Package.

Quast C, Pruesse E, Yilmaz P, Gerken J, Schweer T, Yarza P, Peplies J, Glöckner FO. 2013. The SILVA ribosomal RNA gene database project: improved data processing and web-based tools. *Nucleic Acids Research* 41:D590–D596. doi:10.1093/nar/gks1219

R Core Team. 2020. R: A language and environment for statistical computing. Vienna, Austria: R Foundation for Statistical Computing.

Rognes T, Flouri T, Nichols B, Quince C, Mahé F. 2016. VSEARCH: a versatile open source tool for metagenomics. *PeerJ* 4:e2584. doi:10.7717/peerj.2584

Shannon CE. 1948. A mathematical theory of communication. *Bell System Technical Journal* 27:623–656. doi:https://doi.org/10.1002/j.1538-7305.1948.tb00917.x

Zhang J, Madden TL. 1997. PowerBLAST: a new network BLAST application for interactive or automated sequence analysis and annotation. *Genome Research* 7:649–656. doi:10.1101/gr.7.6.649

Zhu J, Zhao Y, Liu M, Gonzalez-Rivas D, Xu X, Cai W, Qi H, Dai L, Wang Z, Song X, Jiang G, Yang Y. 2019. Developing a new qPCR-based system for screening mutation. *Small* 15:e1805285. doi:10.1002/smll.201805285

**Supplementary Figures and Tables**

**Supplementary Figure 1.** (A) Photograph of unmarked surfaces (UN) and black stains (BS) in the Nave (Source: S. Géraud, DRAC Nouvelle Aquitaine). (B) Map of Lascaux Cave presenting the location studied in September 2020 (Source of the map: S. Konik, Centre National de la Préhistoire). Samples UN1, UN2 and UN3 for unmarked surfaces corresponded to respectively La1299, La1300 and La1301 in the Lascaux database, and samples BS1, BS2 and BS3 for black stains (Kappa classification) to respectively La1294, La1295 and La1296. These black stains formed sometimes between January 2019 and August 2020.

**Supplementary Figure 2.** Distribution of microbial genetic potential in each rock surface condition identified in genomic bins. Each point represents a genomic bin in which the corresponding metabolic class (i.e. Antibiotic biosynthesis, Siderophore biosynthesis, Formaldehyde utilization, Carotenoid biosynthesis, or Melanin precursor and aromatic compounds degradation) has been identified. Details of genomic bins (ID, completeness, contamination, taxonomy and coverage) are presented in Table S3.

**Supplementary Figure 3**. Metabolic profile comparison of two genomic bins affiliated with *Pseudomonas* genus in unmarked surfaces (BIN 1) and black stains (BIN 27). Colors in the inner circles represent (i) pathway class affiliation and (ii) reaction type. Presence of metabolic pathways in BIN 1 (in green) and BIN 27 (in blue) is represented by squares in the outer circle. Details of metabolic pathways are presented in Table S4 (name, class and type of reaction).

**Table S1.** Details of the amplicon and shotgun metagenome sequencing of different samples from Lascaux Cave.

| Sample ID | Sample type | Number of bacterial reads according to amplicon sequencing | Number of archaeal reads according to amplicon sequencing | Number of microeukaryotic reads according to amplicon sequencing | Number of shotgun metagenomic reads | Number of bacterial reads according to shotgun metagenomic sequencing | Number of archaeal reads according to shotgun metagenomic sequencing | Number of microeukaryotic reads according to shotgun metagenomic sequencing |
| --- | --- | --- | --- | --- | --- | --- | --- | --- |
| UN1 | Unmarked surface | 161,176 | 17,388 | 268,819 | 156,652,616 | 133,193,460 | 94,952 | 181,008 |
| UN2 | Unmarked surface | 148,085 | 55,002 | 261,395 | 153,939,324 | 128,194,664 | 328,322 | 111,592 |
| UN3 | Unmarked surface | 196,171 | 127,029 | 297,227 | 166,428,240 | 127,717,260 | 91,074 | 2,230,124 |
| BS1 | Black stain | 146,591 | 15,809 | 380,594 | 151,801,036 | 124,242,664 | 10,866 | 2,659,242 |
| BS2 | Black stain | 127,994 | 11,353 | 275,903 | 167,415,128 | 138,018,498 | 19,806 | 701,898 |
| BS3 | Black stain | 149,097 | 53,549 | 210,384 | 155,391,304 | 137,847,218 | 20,132 | 100,843 |
| Normalization | | 127,994 | 11,353 | 210,384 | 151,801,036 | NA | NA | NA |

**Table S2.** Details of the quality of shotgun metagenome bioinformatic analysis.

| **Characteristics** | **Results** |
| --- | --- |
| Number of contigs | 61968 |
| Total length | 608767283 |
| Longest contig | 1924787 |
| Shortest contig | 2000 |
| N50 | 29699 |
| N90 | 3041 |
| Contigs at superkingdom (k) rank | 59141 (95.4%), in 4 superkingdoms |
| Contigs at phylum (p) rank | 53967 (87.1%), in 29 phyla |
| Contigs at class (c) rank | 44380 (71.6%), in 48 classes |
| Contigs at order (o) rank | 36111 (58.3%), in 89 orders |
| Contigs at family (f) rank | 29352 (47.4%), in 134 families |
| Contigs at genus (g) rank | 23078 (37.2%), in 223 genera |
| Contigs at species (s) rank | 2305 (3.7%), in 219 species |
| Congruent | 57992 (93.6%) |
| Disparity > 0 | 3977 (6.4%) |
| Disparity ≥ 0.25 | 2030 (3.3%) |

| N50: is the minimum contig length to cover 50% of the genome. |
| --- |
| N90: is the minimum contig length to cover 90% of the genome. |
| Congruent means that all genes in the contig belong to the same taxa. |
| Disparity is the percentage of paired comparisons between genes that belong to different taxa. |

**Table S3.** Details of reconstructed bins. UN: Unmarked surface; BS: Black stain.

| BIN ID | Completeness (& contamination) % | Taxonomical identification | | | | Coverage | | | | | | | | |  |
| --- | --- | --- | --- | --- | --- | --- | --- | --- | --- | --- | --- | --- | --- | --- | --- |
|  |  | Kingdom | Phylum | Class | Genus | | UN1 | UN2 | | UN3 | | BS1 | BS2 | BS3 | |
| BIN1 | 89.76 (5.38) | Bacteria | Pseudomonadota | Gammaproteobacteria | *Pseudomonas* | | 19.41 | 7.01 | 34.11 | | 0.00 | | 0.01 | 0.00 | |
| BIN2 | 94.83 (0.05) | Bacteria | Gemmatimonadota | Gemmatimonadia | *Gemmatimonas* | | 12.36 | 4.81 | 17.78 | | 0.00 | | 0.00 | 0.00 | |
| BIN3 | 92.22 (6.86) | Bacteria | Pseudomonadota | Alphaproteobacteria | *Phyllobacterium* | | 36.92 | 25.23 | 0.06 | | 0.00 | | 0.00 | 0.00 | |
| BIN4 | 86.27 (5.38) | Bacteria | Actinomycetota | Thermoleophilia | Not identified | | 3.73 | 55.03 | 10.55 | | 0.00 | | 0.01 | 0.00 | |
| BIN5 | 91.47 (3.42) | Archaea | Nitrososphaerota | Nitrososphaeria | *Candidatus Nitrosocosmicus* | | 0.24 | 6.11 | 59.62 | | 0.00 | | 0.00 | 0.02 | |
| BIN6 | 87.28 (2.37) | Bacteria | Pseudomonadota | Alphaproteobacteria | *Sphingomonas* | | 2.683 | 1.649 | 1.029 | | 38.144 | | 19.310 | 3.243 | |
| BIN7 | 76.70 (3.31) | Bacteria | Bacteroidota | Chitinophagia | *Chitinophaga* | | 2.149 | 2.522 | 5.084 | | 37.807 | | 31.576 | 21.424 | |
| BIN8 | 98.28 (7.03) | Bacteria | Not identified | Not identified | Not identified | | 75.598 | 54.086 | 7.411 | | 36.549 | | 27.361 | 11.710 | |
| BIN9 | 81.82 (0.95) | Bacteria | Pseudomonadota | Gammaproteobacteria | *Xanthomonas* | | 56.519 | 28.749 | 1.198 | | 37.811 | | 39.516 | 22.820 | |
| BIN10 | 90.44 (1.53) | Bacteria | Bacteroidota | Chitinophagia | *Chitinophaga* | | 315.375 | 68.709 | 0.589 | | 37.818 | | 20.959 | 16.865 | |
| BIN11 | 79.54 (2.16) | Bacteria | Pseudomonadota | Betaproteobacteria | Not identified | | 77.236 | 32.132 | 4.553 | | 20.779 | | 16.357 | 6.817 | |
| BIN12 | 76.87 (7.30) | Bacteria | Pseudomonadota | Alphaproteobacteria | *Brevundimonas* | | 623.814 | 965.290 | 984.695 | | 166.449 | | 704.012 | 3.598 | |
| BIN13 | 93.27 (2.62) | Bacteria | Verrucomicrobiota | Verrucomicrobiae | *Akkermansia* | | 118.596 | 43.043 | 6.351 | | 14.528 | | 19.461 | 8.984 | |
| BIN14 | 85.47 (2.17) | Bacteria | Pseudomonadota | Alphaproteobacteria | *Phyllobacterium* | | 29.748 | 26.040 | 4.111 | | 37.820 | | 0.736 | 2.635 | |
| BIN15 | 89.00 (1.98) | Bacteria | Pseudomonadota | Alphaproteobacteria | *Mesorhizobium* | | 8.355 | 5.605 | 5.279 | | 12.963 | | 25.383 | 4.062 | |
| BIN16 | 88.76 (0.80) | Bacteria | Pseudomonadota | Betaproteobacteria | *Advenella* | | 133.199 | 62.680 | 1.758 | | 80.413 | | 17.537 | 11.224 | |
| BIN17 | 92.30 (6.90) | Bacteria | Pseudomonadota | Alphaproteobacteria | *Bradyrhizobium* | | 16.815 | 11.946 | 3.249 | | 6.938 | | 5.724 | 5.172 | |
| BIN18 | 77.51 (1.20) | Bacteria | Pseudomonadota | Alphaproteobacteria | *Agrobacterium* | | 5.091 | 0.016 | 6.541 | | 37.850 | | 13.288 | 3.717 | |
| BIN19 | 86.02(1.45) | Bacteria | Actinomycetota | Actinomycetia | *Nocardioides* | | 10.901 | 0.269 | 0.700 | | 37.816 | | 88.161 | 77.476 | |
| BIN20 | 97.32 (3.19) | Bacteria | Pseudomonadota | Deltaproteobacteria | *Polyangiaceae* | | 100.279 | 41.372 | 1.039 | | 5.320 | | 5.395 | 4.175 | |
| BIN21 | 78.99 (1.13) | Bacteria | Pseudomonadota | Alphaproteobacteria | *Hyphomicrobium* | | 0.00 | 0.00 | 0.00 | | 37.813 | | 82.190 | 27.298 | |
| BIN22 | 85.71 (0.85) | Bacteria | Actinomycetota | Actinomycetia | Not identified | | 0.00 | 0.10 | 0.00 | | 60.390 | | 124.968 | 193.752 | |
| BIN23 | 76.93 (1.71) | Bacteria | Pseudomonadota | Alphaproteobacteria | Not identified | | 0.01 | 0.00 | 0.04 | | 37.817 | | 7.517 | 9.471 | |
| BIN24 | 90.52 (4.25) | Bacteria | Chlamydiota | Chlamydiia | Not identified | | 0.00 | 0.02 | 0.00 | | 14.811 | | 12.635 | 3.392 | |
| BIN25 | 81.47 (5.50) | Bacteria | Bacteroidota | Not identified | Not identified | | 0.03 | 0.00 | 0.00 | | 14.907 | | 31.413 | 54.207 | |
| BIN26 | 80.82 (2.59) | Bacteria | Pseudomonadota | Gammaproteobacteria | *Legionella* | | 0.00 | 0.00 | 0.00 | | 37.809 | | 22.352 | 25.311 | |
| BIN27 | 78.56 (3.82) | Bacteria | Chlamydiota | Chlamydiia | Not identified | | 0.00 | 0.00 | 0.02 | | 37.810 | | 14.210 | 9.526 | |
| BIN28 | 85.59 (2.26) | Bacteria | Bacteroidota | Chitinophagia | *Chitinophaga* | | 0.00 | 0.00 | 0.05 | | 37.814 | | 38.205 | 8.899 | |
| BIN29 | 82.41 (1.69) | Bacteria | Pseudomonadota | Gammaproteobacteria | *Pseudomonas* | | 0.00 | 0.00 | 0.00 | | 37.815 | | 9.641 | 3.480 | |
| BIN30 | 78.57 (1.22) | Bacteria | Actinomycetota | Not identified | Not identified | | 0.311 | 0.00 | 0.00 | | 120.804 | | 156.949 | 41.184 | |
| BIN31 | 88.60 (1.35) | Eukaryota | Ascomycota | Eurotioycetes | *Exophiala* | | 0.00 | 0.00 | 0.02 | | 37.819 | | 131.408 | 19.524 | |

Coverage: average number of sample nucleotide bases aligned to a specific locus in a reconstructed genome.

| **Table S4.** Details of metabolic pathways in *Pseudomonas* bins. | | | |
| --- | --- | --- | --- |
| **Pathway number** | **Metabolic pathway name** | **Type of reaction** | **Pathway class** |
| 1 | cis-dodecenoyl biosynthesis | B | Alcohol |
| 2 | (S,S)-butanediol biosynthesis | B | Alcohol |
| 3 | glycerol degradation I | D | Alcohol |
| 4 | glycerol degradation IV | D | Alcohol |
| 5 | oxidative ethanol degradation III (microsomal) | D | Alcohol |
| 6 | ethanol degradation II (cytosol) | D | Alcohol |
| 7 | (S,S)-butanediol degradation | D | Alcohol |
| 8 | putrescine biosynthesis III | B | Amines and polyamines |
| 9 | spermidine biosynthesis I | B | Amines and polyamines |
| 10 | ureide biosynthesis | B | Amines and polyamines |
| 11 | putrescine biosynthesis II | B | Amines and polyamines |
| 12 | heme biosynthesis from uroporphyrinogen-III II | B | Amines and polyamines |
| 13 | choline degradation I | B | Amines and polyamines |
| 14 | heme biosynthesis from uroporphyrinogen-III I | B | Amines and polyamines |
| 15 | UDP-*N*-acetyl-D-glucosamine biosynthesis I | B | Amines and polyamines |
| 16 | ethanolamine utilization | D | Amines and polyamines |
| 17 | putrescine degradation III | D | Amines and polyamines |
| 18 | urea degradation II | D | Amines and polyamines |
| 19 | urea degradation I | D | Amines and polyamines |
| 20 | choline-*O*-sulfate degradation | D | Amines and polyamines |
| 21 | beta-alanine biosynthesis III | B | Amino acids |
| 22 | alanine biosynthesis I | B | Amino acids |
| 23 | arginine biosynthesis I | B | Amino acids |
| 24 | arginine biosynthesis II (acetyl cycle) | B | Amino acids |
| 25 | aspartate biosynthesis | B | Amino acids |
| 26 | cysteine biosynthesis IV (fungi) | B | Amino acids |
| 27 | glutamate and glutamine biosynthesis | B | Amino acids |
| 28 | glutamate biosynthesis I | B | Amino acids |
| 29 | glutamate biosynthesis III | B | Amino acids |
| 30 | glutamate biosynthesis IV | B | Amino acids |
| 31 | glutamine biosynthesis III | B | Amino acids |
| 32 | glycine biosynthesis III | B | Amino acids |
| 33 | glycine biosynthesis IV | B | Amino acids |
| 34 | histidine biosynthesis | B | Amino acids |
| 35 | homocysteine and cysteine interconversion | B | Amino acids |
| 36 | homoserine biosynthesis | B | Amino acids |
| 37 | isoleucine biosynthesis I (from threonine) | B | Amino acids |
| 38 | isoleucine biosynthesis II | B | Amino acids |
| 39 | isoleucine biosynthesis III | B | Amino acids |
| 40 | isoleucine biosynthesis V | B | Amino acids |
| 41 | leucine biosynthesis | B | Amino acids |
| 42 | lysine biosynthesis I | B | Amino acids |
| 43 | methionine biosynthesis III | B | Amino acids |
| 44 | ornithine biosynthesis | B | Amino acids |
| 45 | superpathway of alanine biosynthesis | B | Amino acids |
| 46 | superpathway of leucine, valine, and isoleucine biosynthesis | B | Amino acids |
| 47 | superpathway of methionine biosynthesis (by sulfhydrylation) | B | Amino acids |
| 48 | superpathway of phenylalanine, tyrosine, and tryptophan biosynthesis | B | Amino acids |
| 49 | alanine biosynthesis II | B | Amino acids |
| 50 | tyrosine biosynthesis IV | B | Amino acids |
| 51 | cardiolipin biosynthesis II | B | Amino acids |
| 52 | cysteine biosynthesis/homocysteine degradation | B | Amino acids |
| 53 | glutamate biosynthesis V | B | Amino acids |
| 54 | superpathway of sulfur amino acid biosynthesis | B | Amino acids |
| 55 | asparagine biosynthesis I | B | Amino acids |
| 56 | glycine betaine biosynthesis I (Gram-negative bacteria) | B | Amino acids |
| 57 | phenylalanine biosynthesis I | B | Amino acids |
| 58 | glycine betaine biosynthesis II (Gram-positive bacteria) | B | Amino acids |
| 59 | cysteine biosynthesis I | B | Amino acids |
| 60 | serine biosynthesis | B | Amino acids |
| 61 | uridine-5'-phosphate biosynthesis | B | Amino acids |
| 62 | glycine cleavage complex | B | Amino acids |
| 63 | tryptophan biosynthesis | B | Amino acids |
| 64 | superpathway of serine and glycine biosynthesis I | B | Amino acids |
| 65 | glycine biosynthesis I | B | Amino acids |
| 66 | L-glutamine biosynthesis II (tRNA-dependent) | B | Amino acids |
| 67 | glutamine biosynthesis I | B | Amino acids |
| 68 | alanine biosynthesis III | B | Amino acids |
| 69 | S-adenosyl-L-methionine cycle II | B | Amino acids |
| 70 | tyrosine biosynthesis I | B | Amino acids |
| 71 | alanine degradation IV | D | Amino acids |
| 72 | arginine degradation II (AST pathway) | D | Amino acids |
| 73 | arginine degradation IX (arginine:pyruvate transaminase pathway) | D | Amino acids |
| 74 | arginine degradation VIII (arginine oxidase pathway) | D | Amino acids |
| 75 | asparagine degradation I | D | Amino acids |
| 76 | aspartate degradation I | D | Amino acids |
| 77 | aspartate degradation II | D | Amino acids |
| 78 | citrulline degradation | D | Amino acids |
| 79 | glutamine degradation I | D | Amino acids |
| 80 | glutamine degradation II | D | Amino acids |
| 81 | histidine degradation II | D | Amino acids |
| 82 | isoleucine degradation I | D | Amino acids |
| 83 | leucine degradation I | D | Amino acids |
| 84 | threonine degradation II | D | Amino acids |
| 85 | alanine degradation III | D | Amino acids |
| 86 | alanine degradation II (to D-lactate) | D | Amino acids |
| 87 | L-cysteine degradation II | D | Amino acids |
| 88 | methionine degradation II | D | Amino acids |
| 89 | arginine degradation IV | D | Amino acids |
| 90 | histidine degradation VI | D | Amino acids |
| 91 | glutamate degradation I | D | Amino acids |
| 92 | histidine degradation I | D | Amino acids |
| 93 | L-serine degradation | D | Amino acids |
| 94 | urate biosynthesis/inosine 5'-phosphate degradation | D | Amino acids |
| 95 | methionine degradation III | D | Amino acids |
| 96 | beta-alanine degradation I | D | Amino acids |
| 97 | beta-alanine degradation II | D | Amino acids |
| 98 | tryptophan degradation to 2-amino-3-carboxymuconate semialdehyde | D | Amino acids |
| 99 | tyrosine degradation I | D | Amino acids |
| 100 | arginine dependent acid resistance | D | Amino acids |
| 101 | *m*-xylene degradation to *m*-toluate | D | Aromatic Compounds |
| 102 | 4-hydroxybenzoate biosynthesis II (bacteria and fungi) | B | Aromatic Compounds |
| 103 | 4-hydroxybenzoate biosynthesis V | B | Aromatic Compounds |
| 104 | 5-aminoimidazole ribonucleotide biosynthesis II | B | Aromatic Compounds |
| 105 | chorismate biosynthesis I | B | Aromatic Compounds |
| 106 | 3-dehydroquinate biosynthesis I | B | Aromatic Compounds |
| 107 | 3-amino-5-hydroxybenzoate biosynthesis | B | Aromatic Compounds |
| 108 | *p*-xylene degradation to *p*-toluate | D | Aromatic Compounds |
| 109 | 2-nitrobenzoate degradation I | D | Aromatic Compounds |
| 110 | 2,4-dinitrotoluene degradation | D | Aromatic Compounds |
| 111 | 3-chloroacrylic acid degradation | D | Aromatic Compounds |
| 112 | 4-aminobutyrate degradation I | D | Aromatic Compounds |
| 113 | 4-aminobutyrate degradation III | D | Aromatic Compounds |
| 114 | benzoate degradation I (aerobic) | D | Aromatic Compounds |
| 115 | ferulate degradation | D | Aromatic Compounds |
| 116 | gentisate degradation | D | Aromatic Compounds |
| 117 | protocatechuate degradation II (ortho-cleavage pathway) | D | Aromatic Compounds |
| 118 | glycolysis III | B | Carbohydrates |
| 119 | pentose phosphate pathway | B | Carbohydrates |
| 120 | pentose phosphate pathway (oxidative branch) | B | Carbohydrates |
| 121 | superpathway of glutamate biosynthesis | B | Carbohydrates |
| 122 | superpathway of glycolysis and Entner-Doudoroff | B | Carbohydrates |
| 123 | Bypass | B | Carbohydrates |
| 124 | trehalose biosynthesis IV | B | Carbohydrates |
| 125 | trehalose biosynthesis V | B | Carbohydrates |
| 126 | UDP-D-galacturonate biosynthesis I (from UDP-D-glucuronate) | B | Carbohydrates |
| 127 | GDP-mannose biosynthesis | B | Carbohydrates |
| 128 | pentose phosphate pathway (non-oxidative branch) | B | Carbohydrates |
| 129 | pentose phosphate pathway (partial) | B | Carbohydrates |
| 130 | dTDP-L-rhamnose biosynthesis I | B | Carbohydrates |
| 131 | dTDP-L-rhamnose biosynthesis II | B | Carbohydrates |
| 132 | Entner-Doudoroff pathway I | B | Carbohydrates |
| 133 | glycolate and glyoxylate degradation I | D | Carbohydrates |
| 134 | sucrose degradation I | D | Carbohydrates |
| 135 | sucrose degradation III | D | Carbohydrates |
| 136 | sucrose degradation IV | D | Carbohydrates |
| 137 | trehalose degradation I (low osmolarity) | D | Carbohydrates |
| 138 | sorbitol degradation I | D | Carbohydrates |
| 139 | D-glucarate degradation I | D | Carbohydrates |
| 140 | 2-amino-3-carboxymuconate semialdehyde degradation to 2-oxopentenoate | D | Carbohydrates |
| 141 | 2-amino-3-carboxymuconate semialdehyde degradation to glutaryl-CoA | D | Carbohydrates |
| 142 | acetate conversion to acetyl-CoA | D | Carbohydrates |
| 143 | acetate formation from acetyl-CoA I | D | Carbohydrates |
| 144 | D-galactarate degradation I | D | Carbohydrates |
| 145 | D-galactarate degradation II | D | Carbohydrates |
| 146 | D-galactonate degradation | D | Carbohydrates |
| 147 | D-glucarate degradation II | D | Carbohydrates |
| 148 | glycogen degradation II | D | Carbohydrates |
| 149 | D-gluconate degradation | D | Carbohydrates |
| 150 | D-mannose degradation | D | Carbohydrates |
| 151 | fructose degradation | D | Carbohydrates |
| 152 | GDP-glucose biosynthesis | D | Carbohydrates |
| 153 | glucose degradation (oxidative) | D | Carbohydrates |
| 154 | glutaryl-CoA degradation | D | Carbohydrates |
| 155 | peptidoglycan biosynthesis I (meso-diaminopimelate containing) | B | Cell Structure |
| 156 | peptidoglycan biosynthesis III (mycobacteria) | B | Cell Structure |
| 157 | UDP-*N*-acetylmuramoyl-pentapeptide biosynthesis III | B | Cell Structure |
| 158 | l,2-Dichloroethane degradation | D | Aromatic compounds |
| 159 | trans, trans-farnesyl diphosphate biosynthesis | B | Cofactors |
| 160 | 6-hydroxymethyl-dihydropterin diphosphate biosynthesis | B | Cofactors |
| 161 | 7-keto-8-aminopelargonate biosynthesis I | B | Cofactors |
| 162 | adenosylcobalamin biosynthesis II (late cobalt incorporation) | B | Cofactors |
| 163 | adenosylcobalamin salvage from cobinamide I | B | Cofactors |
| 164 | biotin biosynthesis from 7-keto-8-aminopelargonate | B | Cofactors |
| 165 | biotin biosynthesis I | B | Cofactors |
| 166 | biotin biosynthesis II | B | Cofactors |
| 167 | flavin biosynthesis I (bacteria and plants) | B | Cofactors |
| 168 | geranyl diphosphate biosynthesis | B | Cofactors |
| 169 | geranylgeranyldiphosphate biosynthesis | B | Cofactors |
| 170 | glutathione biosynthesis | B | Cofactors |
| 171 | heme biosynthesis I | B | Cofactors |
| 172 | heme biosynthesis II | B | Cofactors |
| 173 | heptaprenyl diphosphate biosynthesis | B | Cofactors |
| 174 | lipoate biosynthesis and incorporation I | B | Cofactors |
| 175 | lipoate biosynthesis and incorporation II | B | Cofactors |
| 176 | NAD biosynthesis II (from tryptophan) | B | Cofactors |
| 177 | polyisoprenoid biosynthesis | B | Cofactors |
| 178 | superpathway of geranylgeranyldiphosphate biosynthesis II (via MEP) | B | Cofactors |
| 179 | superpathway of pyridoxal 5'-phosphate biosynthesis and salvage | B | Cofactors |
| 180 | superpathway of tetrahydrofolate biosynthesis and salvage | B | Cofactors |
| 181 | tetrapyrrole biosynthesis I | B | Cofactors |
| 182 | tetrapyrrole biosynthesis II | B | Cofactors |
| 183 | siroheme biosynthesis | B | Cofactors |
| 184 | pantothenate and coenzyme A biosynthesis I | B | Cofactors |
| 185 | phosphopantothenate biosynthesis I | B | Cofactors |
| 186 | formylTHF biosynthesis I | B | Cofactors |
| 187 | adenosylcobalamin salvage from cobalamin | B | Cofactors |
| 188 | coenzyme A biosynthesis | B | Cofactors |
| 189 | factor 43 biosynthesis | B | Cofactors |
| 190 | S-adenosyl-L-methionine biosynthesis | B | Cofactors |
| 191 | menaquinol-6 biosynthesis | B | Cofactors |
| 192 | menaquinol-8 biosynthesis | B | Cofactors |
| 193 | menaquinol-9 biosynthesis | B | Cofactors |
| 194 | thioredoxin pathway | B | Cofactors |
| 195 | gamma-linolenate biosynthesis II | B | Fatty acid and Lipid |
| 196 | biotin-carboxyl carrier protein assembly | B | Fatty acid and Lipid |
| 197 | fatty acid elongation – saturated | B | Fatty acid and Lipid |
| 198 | palmitoleate biosynthesis I | B | Fatty acid and Lipid |
| 199 | fatty acid beta oxidation III (unsaturated, odd number) | B | Fatty acid and Lipid |
| 200 | phospholipid biosynthesis I | B | Fatty acid and Lipid |
| 201 | lipid IV A biosynthesis | B | Fatty acid and Lipid |
| 202 | CDP-diacylglycerol biosynthesis III | B | Fatty acid and Lipid |
| 203 | fatty acid beta-oxidation II (core pathway) | B | Fatty acid and Lipid |
| 204 | phosphatidylglycerol biosynthesis I (plastidic) | B | Fatty acid and Lipid |
| 205 | phosphatidylglycerol biosynthesis II (non-plastidic) | B | Fatty acid and Lipid |
| 206 | cyclopropane and cyclopropene fatty acid biosynthesis | B | Fatty acid and Lipid |
| 207 | cyclopropane fatty acid (CFA) biosynthesis | B | Fatty acid and Lipid |
| 208 | phosphatidylethanolamine biosynthesis I | B | Fatty acid and Lipid |
| 209 | CDP-diacylglycerol biosynthesis I | B | Fatty acid and Lipid |
| 210 | CDP-diacylglycerol biosynthesis II | B | Fatty acid and Lipid |
| 211 | fatty acid activation | B | Fatty acid and Lipid |
| 212 | fatty acid omega oxidation | B | Fatty acid and Lipid |
| 213 | fatty acid biosynthesis initiation I | B | Fatty acid and Lipid |
| 214 | (S)-acetoin biosynthesis | B | Fermentation |
| 215 | heterolactic fermentation | F | Fermentation |
| 216 | formaldehyde oxidation IV (thiol-independent) | O | Formaldehyde |
| 217 | formate oxidation to CO_2_ | O | Formaldehyde |
| 218 | PRPP biosynthesis II | B | Inorganic Nutrients |
| 219 | ammonia assimilation cycle II | D | Inorganic Nutrients |
| 220 | cyanate degradation | D | Inorganic Nutrients |
| 221 | phosphonoacetate degradation | D | Inorganic Nutrients |
| 222 | thiosulfate disproportionation III (rhodanese) | D | Inorganic Nutrients |
| 224 | purine nucleotides *de novo* biosynthesis II | B | Nucleotides |
| 225 | xanthine and xanthosine salvage | B | Nucleotides |
| 226 | superpathway of 5-aminoimidazole ribonucleotide biosynthesis | B | Nucleotides |
| 227 | inosine-5'-phosphate biosynthesis I | B | Nucleotides |
| 228 | pyrimidine ribonucleotides *de novo* biosynthesis | B | Nucleotides |
| 229 | inosine-5'-phosphate biosynthesis II | B | Nucleotides |
| 230 | beta-carboline biosynthesis | B | Secondary Metabolites |
| 231 | betaxanthin biosynthesis (via dopamine) | B | Secondary Metabolites |
| 232 | Biosynthesis of vancomycin group antibiotics | B | Secondary Metabolites |
| 233 | Inositol metabolism | B | Secondary Metabolites |
| 234 | isoprene biosynthesis | B | Secondary Metabolites |
| 235 | methylthiopropionate biosynthesis | B | Secondary Metabolites |
| 236 | tetracycline biosynthesis | B | Secondary Metabolites |
| 237 | valine biosynthesis | B | Secondary Metabolites |
| 238 | methylerythritol phosphate pathway | B | Secondary Metabolites |
| 239 | superpathway of acetyl-CoA biosynthesis | B | Secondary Metabolites |
| 240 | superpathway of rifamycin B biosynthesis | B | Secondary Metabolites |
| 241 | pyoverdine I biosynthesis | B | Siderophores |
